# Human-Mimic Submuscular and Premuscular Irradiated Rat Model: Histologic Characteristics of the Capsule Tissue in Contact with the Breast Implant

**DOI:** 10.1155/2023/4363272

**Published:** 2023-11-09

**Authors:** Hyung Bae Kim, Se Young Han, Jin Sup Eom, Hyun Ho Han

**Affiliations:** Department of Plastic and Reconstructive Surgery, Asan Medical Center, University of Ulsan College of Medicine, Seoul, Republic of Korea

## Abstract

**Background:**

In this study, we established two rat models that mimic human submuscular and premuscular breast reconstruction. We analyzed the capsule formation according to surgical techniques and adjacent tissues, including the chest wall tissues, such as the ribs and acellular dermal matrices (ADMs) that come in contact with silicone implants.

**Methods:**

This study consisted of experiments on 12 Sprague–Dawley rats that underwent implant reconstruction using ADM. They were divided into two groups: rats that underwent dual-plane implantation (*n* = 6; group 1) and those that underwent premuscular implant insertion (*n* = 6; group 2). All rats were irradiated with 35 Gy of fractionated radiation. Three months after surgery, the histology and immunochemistry of the capsule tissues of the ADM, muscle, and chest wall were analyzed.

**Results:**

Overall capsule thickness was thicker in group 1. Based on the tissue in contact with the silicone implant, ADM had a thinner capsule, less inflammation, less fibrosis, and less vascularization than the muscle and chest wall tissues.

**Conclusions:**

This study described two rat models of clinically relevant implant-based breast reconstruction using a submuscular and premuscular plane, ADM, and irradiation. Overall, the premuscular implantation rat model was associated with a thinner capsule. The ADM in contact with the silicone implant, even after irradiation, had superior protection from radiation compared with the other tissues.

## 1. Introduction

All patients planned for mastectomy should be informed of their options for breast reconstruction. Immediate implant-based breast reconstruction is currently the most commonly applied breast reconstruction technique [1, 2]. Adjuvant postmastectomy radiation therapy is administered to reduce the probability of locoregional recurrence but is associated with adverse effects and complications after implant-based breast reconstruction [3, 4]. Capsular contracture is a frequent complication that can affect quality of life and is a risk factor for reoperation and implant failure. A meta-analysis determined capsular contracture to be associated with postmastectomy radiation therapy in 40–50 percent of implant-based breast reconstructions [4].

Various techniques with various planes have been introduced in implant-based reconstruction [5]. The subpectoral plane provides thick soft-tissue coverage with a pectoral muscle flap that can protect the implant. However, there are tradeoffs associated with using the subpectoral plane, such as animation deformity, capsular contracture, and implant migrations [6]. Recently, prepectoral implantation has increased in popularity, with many cited advantages, including less postoperative pain, shorter operation times, and avoidance of animation deformity. Moreover, prepectoral plane implantation alongside acellular dermal matrix (ADM) use may be protective of silicone implants [7, 8]. ADM material is commonly used with implant-based breast reconstruction. Several reports advocate the protective effects of ADMs against capsular contracture, owing to the low vascularity of these matrices. However, controversy remains regarding the selection of ADM-based techniques for breast reconstruction. To determine the relative effectiveness of these surgical methods, it is necessary to know how each type of tissue in contact with the silicone implant would affect the capsule. Clinically analogous small animal models can facilitate an improved understanding of the effects of the tissue and radiation on capsule formation.

Several rodent models for implant-based breast reconstruction have been reported, with various placements of the expander or implant in the dorsal skin and scalp [9]. The dorsal skin of the rat is sufficiently lax for implant placement. The latissimus dorsi muscle could also be used to cover the implant in the dorsal area. In the present study, we designed a rodent model of implant covering with the latissimus dorsi muscle and ADM, which mimics human dual-plane breast reconstruction surgery and implant covering with ADM and skin, which mimics human prepectoral breast reconstruction surgery. With this technique, we hypothesized that the capsule formation varies by a surgical technique and by the types of tissue in contact with silicone implants (muscle tissue; chest wall tissue, including the ribs; and ADM) in irradiated rat models.

## 2. Materials and Methods

### 2.1. Animals

All the animal experiments were performed in accordance with the protocol approved by the Animal Care and Use Committee of our institution (Animal Welfare Assurance no. A20203025). Twelve 8-week-old adult male Sprague–Dawley rats weighing 300–350 g were included. All rats underwent surgery that mimicked human implant-based breast reconstruction. The rats were divided into two groups: the rats in the control group (group 1) underwent submuscular implant-based reconstruction surgery (*n* = 6); group 2 underwent premuscular implant-based reconstruction surgery (*n* = 6). All rats underwent irradiation with 35 Gy of fractionated radiation (five doses of 7 Gy each).

### 2.2. Surgical Techniques

**Figure 1:**
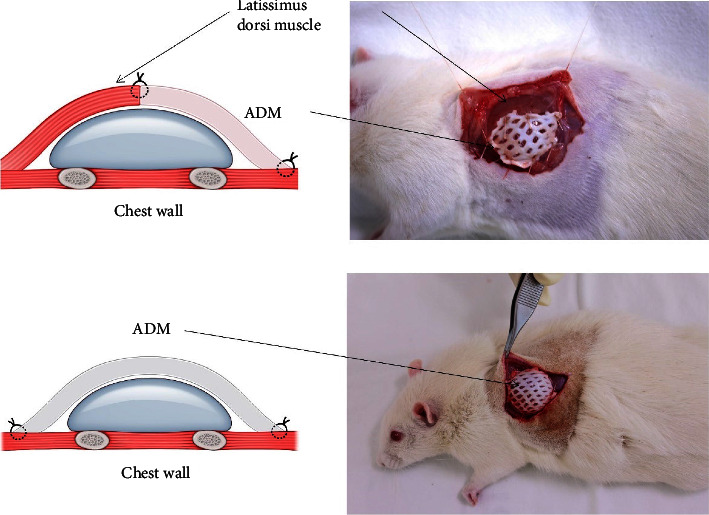
Scheme and intraoperative photos of surgical techniques.

Anesthesia was started and maintained via the inhalation of isoflurane, with a concentration of 2–3 percent. For each rat in group 1, a 3 cm incision was made over the margin of the latissimus dorsi muscle. A submuscular dissection under the latissimus dorsi was performed to form the implant pocket. Then, half of the implant (1.5 × 1.5 cm smooth silicone implant, Hans Biomed, Seoul, Korea) was inserted and covered under the latissimus dorsi. The remnant of the implant-exposed area was covered with porcine ADM (2 × 2 cm, 1.0–2.0 mm thickness, Yejak-Derma Plus, L&C Bio, Seoul, Korea) and securely sutured with absorbable sutures. For each rat in group 2, a 3 cm incision was made at the same site, and the implant was inserted under the skin. The anterior implant surface was covered with ADM and securely sutured with absorbable sutures (Figure 1).

### 2.3. Irradiation

Irradiation was started 2 weeks after surgery. All rats were anesthetized with tiletamine-zolazepam (50 mg/kg) and placed in a small animal irradiator. A 5 mm lead shield was used to protect areas other than the area of implant insertion. All rats received five once-daily doses of 7-Gy irradiation, for a total of 35 Gy. We conducted preliminary experiments to determine the reasonable dose of radiation and found that a total dose of 35 Gy of fractional radiation was safe and yielded sufficient histologic changes. Moreover, 45 Gy of fractional radiation was fatally toxic to the rats.

### 2.4. Histology

All of the rats were sacrificed 3 months after surgery. The capsule and pericapsular tissues were harvested from the ADM, latissimus dorsi muscle tissue, and chest wall tissue. The hematoxylin and eosin-stained sections were analyzed for capsule thickness and inflammatory cell counts. Immunohistochemistry evaluation was performed to indicate myofibroblast and endothelial cell presence using an alpha-smooth muscle actin (*α*-SMA) monoclonal antibody (1A4 Mouse Monoclonal Antibody, Merck KGaA, Darmstadt, Germany) and a monoclonal antibody of CD31 (Anti-CD31 Monoclonal Antibody, Abcam, Cambridge, UK), respectively. A researcher (SY Han) in the current study's department was in charge of randomly selecting histological fields. The first author (HB Kim) conducted the analysis; however, random numbering was used to reduce observer bias.

Histologic analysis of the capsule formation was performed with two approaches. One approach was to analyze the capsule formation according to the implant insertion plane, and the other approach was to analyze the capsule formation in contact with the muscle tissue, ADM, and chest wall tissue.

### 2.5. Capsule Thickness

The hematoxylin and eosin-stained sections were analyzed for capsule thickness and the inflammatory cell count. Capsule thickness was measured by averaging the values at the center of three separate images (×100 magnification).

### 2.6. Inflammatory Cell Count

Using the hematoxylin and eosin-stained sections, we counted the number of nucleated fibroblasts and inflammatory cells in the capsule at high magnification (×400 magnification).

### 2.7. Immunohistochemistry of *α*-SMA

The immunohistochemistry analysis for myofibroblast detection was carried out via *α*-SMA staining (×400 magnification). The percentage of the stained area in the capsule (×400 magnification) was calculated using ImageJ, version 1.52 (National Institutes of Health, Bethesda, MD, USA) via the segmentation of the immunostained area.

### 2.8. Immunohistochemistry of CD31

Immunohistochemistry for endothelial cells was determined using CD31. The CD31-stained vessels were counted around the capsule and pericapsular tissue at high magnification (×100 magnification).

### 2.9. Statistical Analysis

Statistical analysis was performed using SPSS Statistics for Windows, version 21 (IBM Corp., Armonk, NY, USA). Pearson's correlation coefficient was used to analyze the correlation between capsule thickness and inflammation, myofibroblast activity, and vascularity. The data were compared with the Mann–Whitney *U* test. A *p* value < 0.05 was considered statistically significant.

## 3. Results

All of the rats survived the 35 Gy of irradiation, and no postoperative complications associated with implantation or irradiation were observed.

### 3.1. Histologic Results According to the Implantation Plane (Submuscular versus Premuscular)

#### 3.1.1. Capsule Thickness

**Figure 2:**
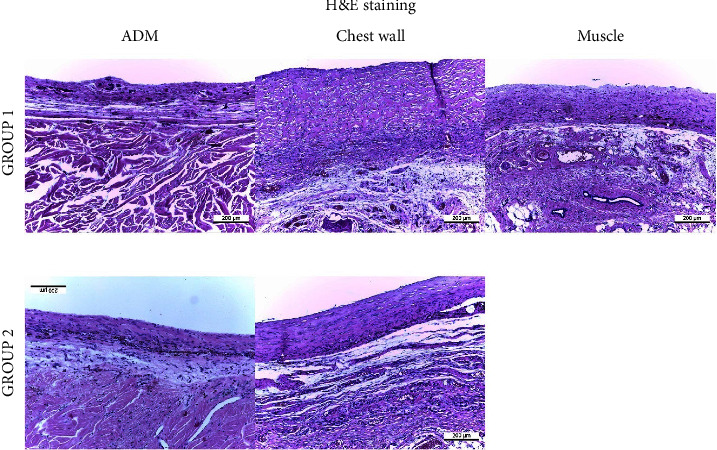
Histological examples of the capsule tissue (hematoxylin and eosin).

**Figure 3:**
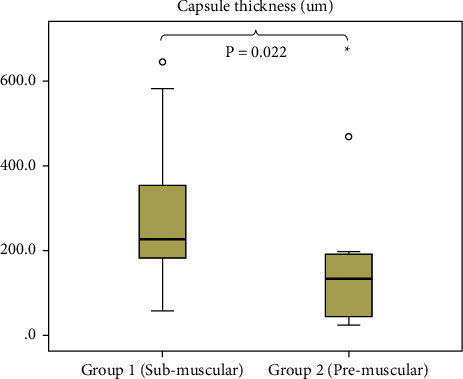
Assessment of capsule thickness according to the surgical technique.

Through hematoxylin and eosin staining (Figure 2), the capsule thickness was measured for each group. The median capsule thickness according to the radiation dose was 227.0 *μ*m (interquartile range (IQR), 192.0 *μ*m) in group 1 compared with 133.0 *μ*m (IQR, 158.5 *μ*m) in group 2. The capsule thickness was significantly higher in the submuscular implantation plane (Figure 3).

#### 3.1.2. Inflammatory Cell Counts

**Figure 4:**
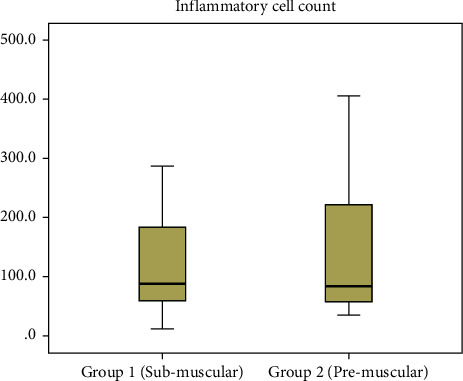
Assessment of the inflammatory cell count according to the surgical technique.

The median inflammatory cell count according to the implantation plane was 87.0 (IQR, 126.5) in group 1 compared with 84.0 (IQR, 180.0) in group 2 (*p*=0.884) (Figure 4).

#### 3.1.3. Immunohistochemistry of *α*-SMA

**Figure 5:**
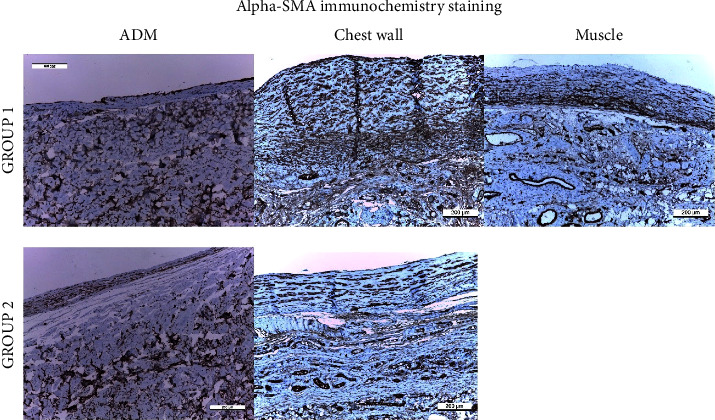
Immunohistochemistry of *α*-SMA.

**Figure 6:**
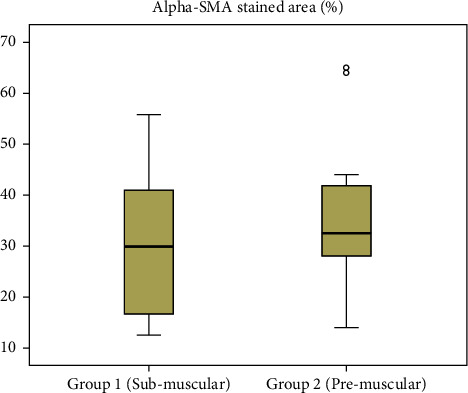
Assessment of the percentage of the *α*-SMA-stained area according to the surgical technique.

Immunohistochemical detection of *α*-SMA was performed in capsule tissues (Figure 5). The median percentage of the *α*-SMA-stained area was 29.7 percent (IQR, 25.4 percent) in group 1 compared with 32.5 percent (IQR, 16.5 percent) in group 2 (*p*=0.518) (Figure 6).

#### 3.1.4. Immunohistochemistry of CD31

**Figure 7:**
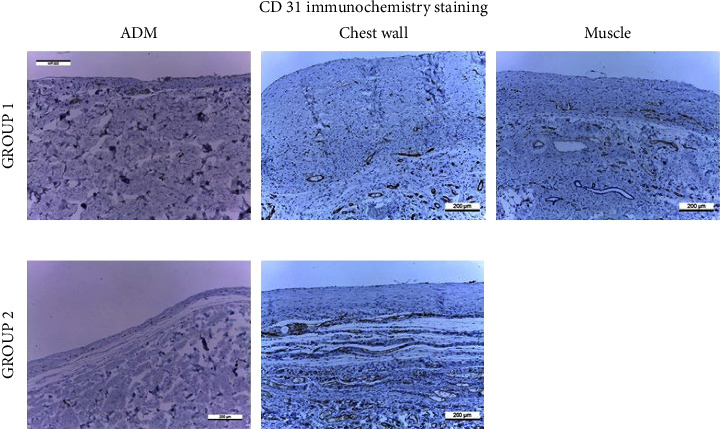
Immunohistochemistry of CD31.

**Figure 8:**
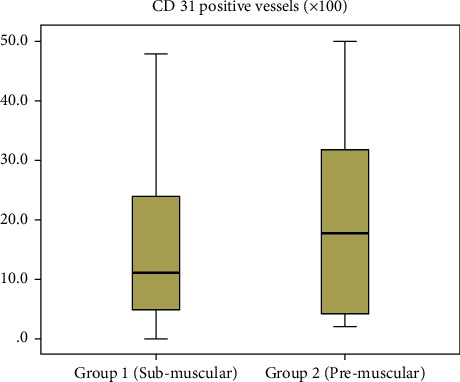
Assessment of the CD31-stained vessel count according to surgical techniques.

Immunohistochemical detection of CD31 was performed in capsule tissues (Figure 7). The median number of CD31-stained vessels was 11.0 (IQR, 20.8) in group 1 compared with 16.0 (IQR, 26.5) in group 2 (*p*=0.884) (Figure 8).

### 3.2. Histologic Results of the Muscle Tissue, ADM, and Chest Wall Tissue

#### 3.2.1. Capsule Thickness

**Figure 9:**
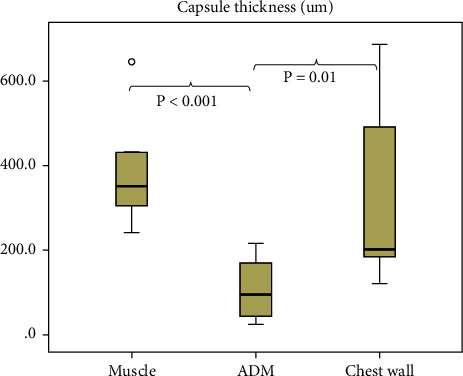
Assessment of capsule thickness according to tissue types.

Through hematoxylin and eosin staining, the capsule thickness was measured in the ADM, muscle tissue, and chest wall tissue. The median capsule thickness was 351.0 *μ*m (IQR, 196.0 *μ*m) in the muscle, 95.0 *μ*m (IQR, 137.0) in ADM, and 200.5 *μ*m (IQR, 321.3 *μ*m) in the chest wall. The capsule in ADM was significantly thinner than that in the muscle or chest wall tissue (vs. muscle, *p* < 0.001; vs. chest wall, *p*=0.01) (Figure 9).

#### 3.2.2. Inflammatory Cell Counts

The median inflammatory cell count was 149.0 (IQR, 170.8) in the muscle tissue, 56.0 (IQR, 27.8) in ADM, and 142.0 (IQR, 146.5) in the chest wall tissue. The inflammatory cell count of ADM was significantly lower than that of the muscle or chest wall tissue (vs. muscle, *p* < 0.001; vs. chest wall, *p*=0.01).

#### 3.2.3. Immunohistochemistry of *α*-SMA

The median percentage of the *α*-SMA stained area was 42.7 percent (IQR, 11.4 percent) in the muscle tissue, 25.0 percent (IQR, 15.5 percent) in ADM, and 36.1 percent (IQR, 21.3 percent) in the chest wall tissue. The median percentage of *α*-SMA in ADM was significantly lower than that in the muscle or chest wall tissue (vs. muscle, *p*=0.002; vs. chest wall, *p*=0.001).

#### 3.2.4. Immunohistochemistry of CD31

The median number of CD31-stained vessels was 27.5 (IQR, 28.5) in the muscle tissue, 4.5 (IQR, 4.3) in ADM, and 26.0 (IQR, 16.5) in the chest wall tissue. The median CD31-stained vessel count in ADM was significantly lower than that in the muscle or chest wall tissue (vs. muscle, *p*=0.003; vs. chest wall, *p* < 0.001).

### 3.3. Correlation between Capsule Thickness and Inflammation, Activity of Myofibroblast, and Vascularity

**Figure 10:**
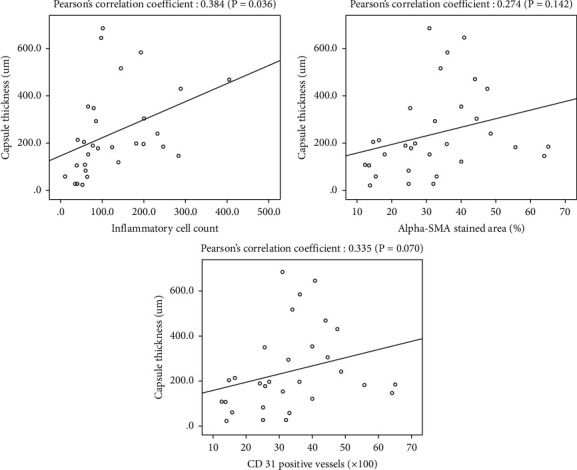
Correlation between capsule thickness and the inflammatory cell count, the percentage of the *α*-SMA-stained area, and the CD31-stained vessel count.

A statistically significant positive correlation was observed between capsule thickness and the inflammatory cell count (correlation coefficient, 0.384; *p*=0.036). Nonsignificant positive correlations were observed between capsule thickness and percentage of the *α*-SMA stained area (correlation coefficient, 0.274; *p*=0.142), as well as between capsule thickness and the CD31-stained vessel count (correlation coefficient, 0.335; *p*=0.070) (Figure 10).

### 3.4. Comparison of the Capsule Characteristics between the Submuscular and Premuscular Groups (Subgroup Analysis)

For capsule characteristics under ADM, capsule thickness was lower in the premuscular group and percentage of the *α*-SMA stained area and the CD31-stained vessel count were lower in the submuscular group. For capsule characteristics under the chest wall, the inflammatory cell count was lower in the submuscular group (Table 1).

## 4. Discussion

This study described two rat models of clinically relevant implant-based breast reconstruction techniques using submuscular and premuscular implantation planes and ADM with adjuvant irradiation. Several studies have investigated rat models of implant-based breast reconstruction followed by postmastectomy radiation therapy [9, 10]. The present study investigated the application of ADM and muscle flaps to mimic clinically relevant submuscular and premuscular implant-based breast reconstruction. Our surgical techniques, which modeled subpectoral and prepectoral implantation, were safe and stably maintained after irradiation. We observed that capsule formation was consistent and comparable between groups and among various tissues. A strength of this study was that, by harvesting capsules from various irradiated tissues, various effects on the capsules were observed and evaluated.

Capsular contracture has been a challenge and has been among the common complications associated with breast implant insertion since breast implant surgery became commonplace in the early 1960s [11]. Despite extensive evidence and clinical experience, the exact pathophysiologic mechanisms underlying capsular contracture remain unknown, and attempts at preventive therapies have resulted in unfavorable results. In clinical settings, it is hard to evaluate the capsule tissue directly while maintaining control of modifiable factors. We aimed to establish models of clinical breast reconstruction and capsule formation in various histologic analyses.

Various techniques with various implantation planes have been introduced in implant-based reconstruction [5, 12, 13]. The plane of implant placement is currently a hot topic. There is no consensus on the ideal implantation plane in breast reconstruction. A meta-analysis found that, compared with a subpectoral plane, a prepectoral implantation plane was significantly associated with reduced capsular contracture (OR, 0.45; 95% confidence interval, 0.27–0.73) [5]. However, there is no consensus regarding the superiority of either prepectoral or subpectoral implantation in breast reconstruction in terms of capsular contracture avoidance.

In our study, overall, capsule thickness was lower in association with premuscular implantation. However, inflammation, myofibroblast activity, and vascularity were comparable between the two groups. Our results indicate that the choice of a surgical technique did not affect the occurrence of inflammation, myofibroblast activity, or vascularity. It seems that the thinner capsule formation associated with premuscular implantation is affected by an absence of muscle tissue. After radiation injury, muscle tissue is known to have an inflammatory reaction that leads to fibrosis and contracture [14]. Muscular contractions may mechanically irritate the implant, thus promoting capsule formation. Another consideration is that, compared with submuscular implantation, premuscular implantation requires more ADM to cover the entire anterior surface. Given that ADM was protective against capsular contracture in this study, premuscular implantation may be associated with reduced capsule formation because of the more extensive use of ADM.

Myofibroblasts are contractile fibroblasts that play a critical role in wound healing, as well as in the capsule formation associated with implant-based breast surgery [10, 14, 15]. Immunohistochemistry staining for *α*-SMA, a marker of differentiated myofibroblasts, is used to localize myofibroblasts near the capsule tissue. Differentiated myofibroblasts are capable of generating high contractile forces [14]. In the present study, the choice of a surgical technique did not significantly affect myofibroblast activity. ADM tissue had fewer myofibroblasts in capsule tissue compared with the other tissue types. These results indicate that ADM is protective against capsular contracture by inhibiting myofibroblast formation.

In the present study, ADM was associated with thinner capsule formation, less inflammation, less fibrosis, and less vascularity. These results are indicative of a protective effect garnered by ADM against capsular contracture. The hypothesis that ADM prevents capsular contracture by blocking these inflammatory responses has been supported by many studies. In another study that evaluated the capsule formation associated with two-stage breast reconstruction, investigators determined low myofibroblast counts in the capsule with ADM, and they observed thin capsules [16]. Furthermore, animal studies on implantation with ADM have found that ADM decreases radiation-related inflammation and slows the progression of capsular contracture [17].

In a pilot study that is not described in this article, we also designed a total-wraparound implant rat model. However, total implant wrapping was not easy because of thick and stiff porcine ADM. Immediate wound closure was also challenging because of the increased volume of the totally wrapped implant. Using human-derived ADM for animal experimentation is illegal in our country. If total implant wrapping was possible, we hypothesize that there would be significant differences between the surgical techniques in terms of inflammation, myofibroblast activity, and vascularity.

In the present study, capsule thickness positively correlated with inflammation. Vascularity and *α*-SMA staining also yielded (nonsignificant) positive correlations with inflammation. One plausible explanation regarding the pathogenesis of capsular contracture is based on the local inflammatory response that is the first pathogenic mechanism in capsule formation [18]. A nonspecific inflammatory process is created around the breast implant, which leads to inflammatory cell infiltration and, eventually, capsule fibrosis and shrinking. Our results—particularly the correlation between capsule thickness and inflammation—support this hypothesis.

Our study's rat model of breast reconstruction mimics the submuscular and premuscular implantation using ADM in human breast reconstruction. The ideal anatomical placement of the breast implant has long been controversial. Total muscle coverage of the implant was introduced to minimize the risk of exposure of the implant in the case of mastectomy skin flap necrosis [19]. However, negative surgical outcomes, such as animation deformity and pain due to muscle spasms, have encouraged plastic surgeons to use other implantation methods. The introduction of ADM in 2006 was a paradigm shift in implant insertion. Partial muscle coverage using ADM became popular because it provides better breast projection and inferolateral tissue support [20]. With the innovations in ADM production, fat grafting, and tissue perfusion technology, prepectoral implantation has become a popular option in breast reconstruction [12, 13]. Recently, several clinical studies have found prepectoral implant placement in breast reconstruction to be associated with lower rates of capsular contracture [5, 21]. The thinner capsules associated with premuscular implant insertion and ADM in this study may support the hypothesis that prepectoral breast implant reconstruction may be associated with a lower incidence of capsular contracture.

This study had some limitations. The postoperative evaluation time (3 months) was relatively short, and capsular contracture can occur any time after implantation. A long-term observational study is warranted to investigate the long-term effects of radiation on capsular contracture. The small sample size of 12 rats was also a limitation. Implantation away from the breast tissue should also be considered. However, given the anatomical composition of rats, implant placement under the chest area is difficult due to the mechanical stress that it would cause and the small chest cavity volume. Inflammation was evaluated by the inflammatory cell count in this study. It could be a better study when we use the inflammatory cytokine rather than inflammatory cell count for evaluating the capsule inflammation.

## 5. Conclusion

We established two rat models of human implant-based submuscular and premuscular breast reconstruction surgery using ADM followed by adjuvant radiation therapy. Premuscular implant placement was associated with thinner capsule formation compared with dual placement. In terms of the tissue in contact with the silicone implant, ADM was associated with thinner capsule formation, less inflammation, less myofibroblast activity, and less vascularity than the muscle and chest wall tissues. Our findings indicate that ADM in contact with the silicone implant, even after irradiation, was protective against radiation toxicity compared with the other tissues (muscle and chest wall) investigated.

## Figures and Tables

**Table 1:** Comparison of the capsule characteristics between the submuscular and premuscular groups.

	Submuscular	Premuscular	*p* value
Capsule in ADM			
Capsule thickness (*μ*m)	143.8 ± 61.5	62.0 ± 50.5	0.026
Inflammatory cell count	70.1 ± 60.6	62.8 ± 29.8	0.937
*α*-SMA (%)	10.0 ± 5.4	29.5 ± 9.3	0.004
CD31 vessels	1.17 ± 0.9	5.0 ± 2.7	0.004
Capsule in the chest wall			
Capsule thickness (*μ*m)	328.3 ± 177.6	300.17 ± 226.8	0.485
Inflammatory cell count	113.1 ± 50.6	228.8 ± 109.2	0.041
*α*-SMA (%)	32.9 ± 13.6	46.6 ± 14.4	0.132
CD31 vessels	20.8 ± 14.9	32.5 ± 9.5	0.065

## Data Availability

No data were used in this study.
